# Population Structure and Genetic Diversity of Native and Invasive Populations of *Solanum rostratum* (Solanaceae) 

**DOI:** 10.1371/journal.pone.0079807

**Published:** 2013-11-05

**Authors:** Jiali Zhao, Lislie Solís-Montero, Anru Lou, Mario Vallejo-Marín

**Affiliations:** 1 State Key Laboratory of Earth Surface Processes and Resource Ecology, College of Life Sciences, Beijing Normal University, Beijing, China; 2 Biological and Environmental Sciences, School of Natural Sciences, University of Stirling, Stirling, United Kingdom; Agriculture and Agri-Food Canada, Canada

## Abstract

**Aims:**

We investigate native and introduced populations of *Solanum rostratum*, an annual, self-compatible plant that has been introduced around the globe. This study is the first to compare the genetic diversity of *Solanum rostratum* between native and introduced populations. We aim to (1) determine the level of genetic diversity across the studied regions; (2) explore the likely origins of invasive populations in China; and (3) investigate whether there is the evidence of multiple introductions into China.

**Methods:**

We genotyped 329 individuals at 10 microsatellite loci to determine the levels of genetic diversity and to investigate population structure of native and introduced populations of *S. rostratum*. We studied five populations in each of three regions across two continents: Mexico, the U.S.A. and China.

**Important Findings:**

We found the highest genetic diversity among Mexican populations of *S. rostratum*. Genetic diversity was significantly lower in Chinese and U.S.A. populations, but we found no regional difference in inbreeding coefficients (*F*_IS_) or population differentiation (*F*_ST_). Population structure analyses indicate that Chinese and U.S.A. populations are more closely related to each other than to sampled Mexican populations, revealing that introduced populations in China share an origin with the sampled U.S.A. populations. The distinctiveness between some introduced populations indicates multiple introductions of *S. rostratum* into China.

## Introduction

Studying the genetic diversity and structure of introduced populations is a key component to understand the potential of introduced species to establish and spread in the novel range [1,2]. For example, severe reductions in genetic diversity may limit the ability of introduced populations to quickly adapt to novel environmental conditions as rapid evolution in invasive species is expected to occur from existing genetic variation [3]. Nevertheless, there are examples of successful invasive species in the absence of significant amounts of neutral genetic diversity [4,5], and even small populations may maintain genetic variation in quantitative traits [6]. In contrast, multiple introductions may be important not only for maintaining variation in introduced populations [7], but also in bringing about novel combinations of genetic variation not seen in the native range. It is thus not surprising that investigating the population genetics of invasive species continues to be at the heart of many ecological, evolutionary and conservation studies.

*Solanum rostratum* is a self-compatible species that produces nectarless hermaphroditic flowers with dimorphic anthers, and is pollinated by bees [8]. Individual plants can grow to approximately 1m in height and produce up to 1915 fruits (72.7± 52.2; mean ± SD, range: 12-1915), each containing an average of 41 seeds; single plants have been recorded to produce in excess of 78000 seeds [9]. Thought to be originated from a region centred on the Mexican highlands [10,11], *Solanum rostratum* has spread to the U.S.A., Canada [12], Europe, Australia [13], the former Soviet Union [10], South Korea, and China [14,15]. In many of these areas, *S. rostratum* is treated as a noxious weed as it grows aggressively following habitat disturbance [15,16], and livestock is discouraged from grazing on vegetation where it grows as thorns cover all the plant except the flowers and can cause poisoning if ingested [16].

One of the most recent invasions of *S. rostratum* has occurred in China during the last 30 years, with the first record made in ChaoYang city of the Liaoning province in 1981 [17]. Despite being a relatively new arrival, *S. rostratum* has spread across a large area in northern China, namely in the provinces of Beijing, Hebei, Jilin, Liaoning, Shanxi provinces and Xinjiang Uygur Autonomous Region [9,18-20]. As in other invaded regions, Chinese populations of *S. rostratum* usually grow in open, disturbed habitats, such as roadsides, fallow fields and along train tracks. Previous studies have shown on-going dispersal towards the north of China, and indicate that *S. rostratum* is at potential risk of an outbreak [13].

We use recently developed genetic tools [21] to investigate the genetic diversity and population structure of native and introduced populations of *S. rostratum*. We studied five populations in each of three regions across two continents: Mexico, the U.S.A. and China. We genotyped individuals using 10 microsatellite loci to address the following questions: (1) What is the level of genetic diversity across the studied regions and to what extent is genetic diversity reduced in introduced populations? (2) What are the likely sources of origin of invasive populations in China inferred from the genetic relationships among samples? (3) Is there evidence of multiple introductions into China? Our study represents the first attempt to characterise the genetic diversity and population structure of native and introduced populations of *S. rostratum* and offers a unique insight into the historical pathways of dispersal of this invasive weed*.*

## Materials and Methods

### Population sampling

**Figure 1:**
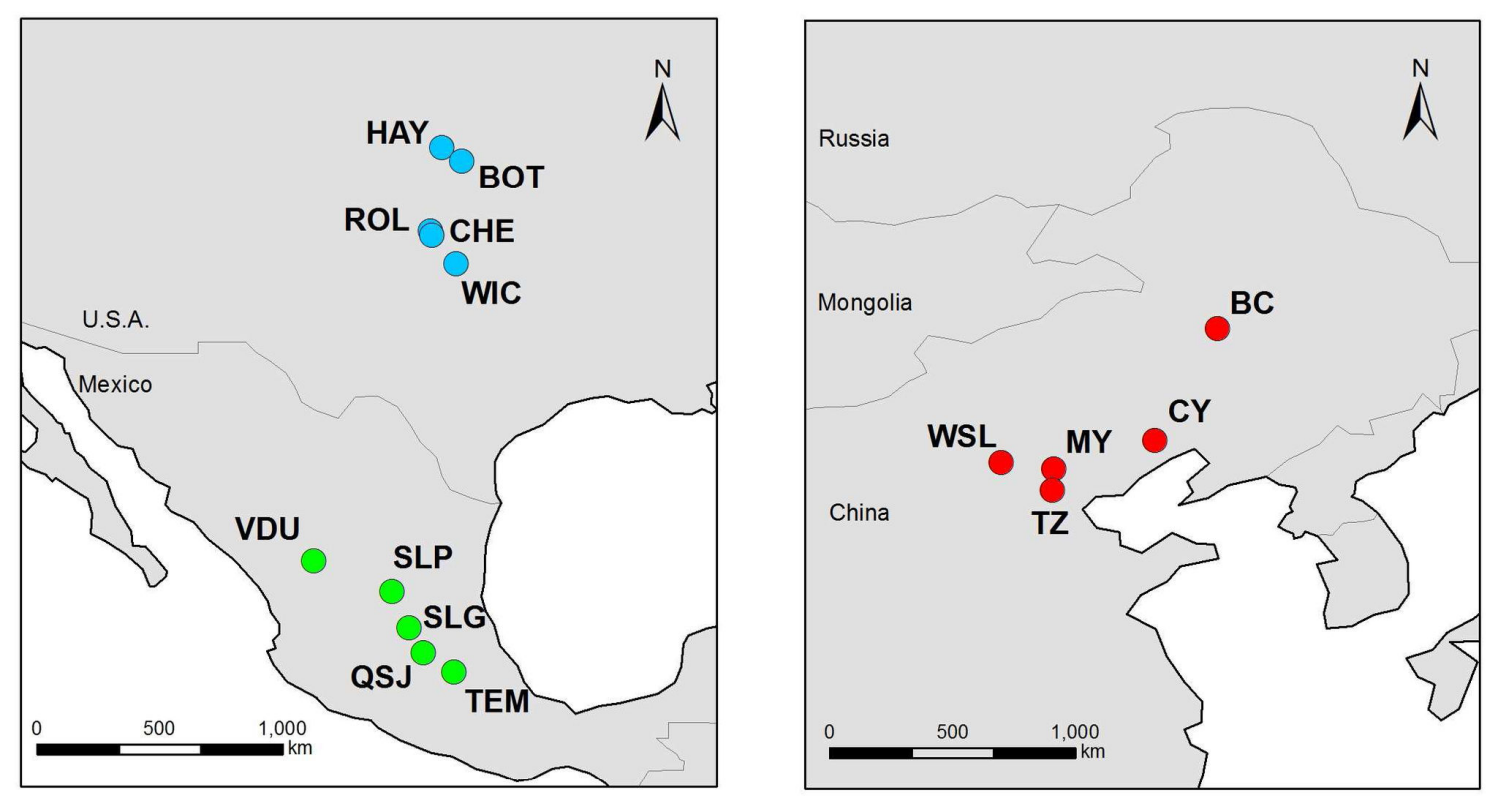
Map showing the location of the 15 sampled populations of *Solanum rostratum* used in this study. Left panel: Mexican (native, green circles) and U.S.A. populations from the U.S.A. (residence time >130 years, blue circles). Right panel: Chinese populations (residence time <31 years, red circles). Details of localities and population codes are provided in Table 1.

We randomly collected fresh leaves of from individuals of *Solanum rostratum*. The sampling sites were located in road sides, banks of rivers, waste land. These sites didn't belong to a national park or other protected area of land and the relevant regulatory body concerned with protection of wildlife, and they also didn't belong to private land. We confirm that the field studies did not involve endangered or protected species. A total of 15 populations were sampled from July 2010 to June 2011. Five populations were sampled from each of the following three regions: central Mexico, the Kansas-Oklahoma region in the U.S.A. where records date back at least to the 1880's (Kansas State University Herbarium) and northern China (Table 1, Figure 1). These three regions were chosen to represent a range of residence histories for *S. rostratum* from the native range in Mexico, to the Kansas-Oklahoma region where *S. rostratum* has been reported for at least 130 years, to the recent introduction of this species to China in the last 30 years.

**Table 1:** Localities and number of individuals successfully genotyped for each of the 15 populations analysed in this study.

**Code**	**Region and Population**	**State or Province**	**Latitude**	**Longitude**	**Altitude (m)**	**Individuals genotyped**
	**China**					
**BC**	Baicheng	Jilin	45.352°	122.501°	154	24
**CY**	Chaoyang	Liaoning	41.273°	120.186°	190	23
**WSL**	Zhangjiakou	Hebei	40.454°	114.552°	729	24
**MY**	Miyun	Beijing	40.239°	116.504°	85	24
**TZ**	Tongzhou	Beijing	39.451°	116.435°	17	24
	**U.S.A.**					
**HAY**	Hays	Kansas	38.915°	-99.315°	645	24
**BOT**	Cheyenne Bottoms	Kansas	38.423°	-98.573°	548	24
**ROL**	Roll	Oklahoma	35.836°	-99.730°	677	24
**CHE**	Cheyenne	Oklahoma	35.675°	-99.679°	614	24
**WIC**	Wichita (Snyder)	Oklahoma	34.631°	-98.791°	403	24
	**Mexico**					
**VDU**	Vicente Guerrero	Durango	23.744°	-103.996°	1926	19
**SLP**	San Antonio del Rul	San Luis Potosí	22.640°	-101.140°	1873	15
**SLG**	San Luis de la Paz	Guanajuato	21.310°	-100.514°	2050	22
**QSJ**	San Juan del Río	Querétaro	20.377°	-99.993°	1955	16
**TEM**	San Juan Teotihuacán	Estado de México	19.684°	-98.859°	2277	18

### Sample preparation and genotyping

In each population, fresh leaves were collected from 20—30 randomly chosen individuals and quickly dried in plastic or paper bags with silica gel. Between September and November 2011, DNA was extracted from dried leaves using TIANGEN plant genomic DNA kit (Tiangen Biotech, Beijing, China) following manufacturer’s instructions. Individuals were genotyped at ten microsatellite loci previously developed for *S. rostratum* [21]. Estimates of allelic dropout, false alleles, and null allele frequencies for a sample of Mexican populations are given in Vallejo-Marín et al. [22]. All loci were amplified in a multiplex PCR using QIAGEN Type-it Microsatellite PCR Kit (Qiagen, Shanghai, China), 100µM concentration of each primer (labelled with one of 6-FAM, VIC, PET, or NED fluorescent dyes; Life Technologies, Shanghai, China), and DNA template. PCR program was as follows: one cycle of 95°C for 5min, 30 cycles of 95°C for 30s, 58°C for 180s, and 72°C for 30s, followed by a final step at 60°C for 30min. Fragment analysis of PCR products was done using an ABI3730xl capillary sequencer with a 80-500bp size standard. Fluorescent profiles were analysed and binned in GeneMapper V3.0 (Applied Biosystem 2002, Foster City, CA, USA). Data are available from the Dryad Digital Repository: http://dx.doi.org/10.5061/dryad.mk58d.

### Genetic Analysis

#### Genetic variation

For each population-locus combination, the following indices were calculated using GENALEX 6.4 [23]: number of alleles (*N*_a_); number of effective alleles (*N*_e_); expected heterozygosity (*H*_E_); observed heterozygosity (*H*_O_); inbreeding coefficient (*F*_IS_). In addition, for each population we also calculated the proportion of polymorphic loci (*P*), allelic richness averaged across loci (*R*_S_), and the number of private alleles. The significance of *F*_IS_ was calculated using 150000 randomizations, and adjusting the significance threshold for multiple comparisons using a Bonferroni correction in FSTAT 2.9.3.2 [24].

To test for overall differences in genetic variation between the three geographic regions (Mexico, the U.S.A. and China), we compared allelic richness, *H*_O_, *H*_E_, *F*_IS_ and *F*_ST_, and relatedness using 1000 permutations in FSTAT [24]. We also tested the hypothesis that populations in the U.S.A. and China have lower levels of genetic variation than Mexican populations using a one-tailed test based on 1000 permutations.

#### Population Genetic Structure

To assess the level of population genetic structure we used a series of complementary approaches. We calculated pairwise *F*_ST_ using FSTAT, and used these statistics to compare the average levels of between-population differentiation in the three studied regions. In addition, we calculated pairwise genetic distances between populations using Nei’s (1978) standardized genetic distance *Ds*. We used the resulting distance matrix to generate an UPGMA tree using TFPGA1.3 [25], and assessed the support for each node using 1000 bootstrap replicates implemented in POPULATIONS 1.2.30 [26].

We also used an analysis of molecular variance (AMOVA, [27]) implemented in Arlequin 3.5.1.3 [28] to partition genetic variation across nested levels: within populations, between populations within geographic regions, and between geographic regions. For the AMOVA, we used the number of different alleles as a measure of genetic variation (F_ST_-like option in Arlequin) and 1000 permutations to test for statistical significance.

In addition, we used InStruct [29] to jointly assign individuals to groups and calculate inbreeding coefficients per population. InStruct is similar to the program STRUCTURE, in that it uses a multi-locus clustering method to probabilistically assign individuals to *K* groups that minimize the amount of linkage-disequilibrium [30]. Because the assignment of each individual to a given group is done probabilistically, this approach can detect admixed or migrant individuals. Importantly, a difference between STRUCTURE and InStruct is that the latter allows for inbreeding to occur within populations. Incorporating inbreeding in this analysis is important as *S. rostratum* has a mixed mating system in both native and introduced populations ([22]; L. Yu and A. Lou *unpublished data*). The InStruct analysis was conducted in a nested fashion. We first analysed the entire data set to determine the number of clusters across the three geographic regions. We ran InStruct with *K*-values between 1 and 15 (the number of studied populations), using a 100000 burn-in and 100000 MCMC iterations with trimming every 100 generations, and estimating both admixture and cluster's selfing rate. Each MCMC chain was re-run three times. Because accurately determining the optimal number of clusters can be difficult when regions display strong genetic structure, we then ran InStruct separately for the populations in the two identified clusters (see Results): Mexico (*K*-values 1—5) and USA/China (*K*-values 1—10), using the same parameter values. The optimal number of clusters (K*) for each analysis was assessed using *∆K* statistic [31].

Finally, we used graph theory implemented in POPULATION GRAPH [32] to determine the minimum number of edges (connections) between populations. This approach uses the genetic data to determine the covariance relationships among populations independently of the geographic region of origin, and has been used in similar population genetic analyses [33,34]. An advantage of this method is that it does not rely on specific assumptions about Hardy-Weinberg equilibrium within populations or clusters [32]. 

## Results

### Genetic diversity across geographic regions

A total of 329 individuals belonging to 15 populations in Mexico, the U.S.A. and China were successfully genotyped using the panel of 10 microsatellite loci. A full description of genetic diversity parameters per locus per population is provided in Table S1. On average, the proportion of polymorphic loci across populations was high (0.95 ± 0.03; mean ± S.E.), while the average number of alleles per locus per population was relatively low (3.17 ± 0.20; for 21.93 ± 0.84 individuals genotyped per population) (Table 2). Two out of five populations in both China and Mexico had at least one monomorphic locus, while only one of five US populations had a single monomorphic locus (WIC; Table 2).There was a deficit in the observed number of heterozygotes across populations (Table 2), indicative of an average inbreeding coefficient of *F*_IS_= 0.252 ± 0.038. Individual populations showed a broad range of inbreeding levels, particularly in China where *F*_IS_ varied widely (0.023—0.521) but also in the U.S.A. (*F*_IS_ = 0.104—0.433) and to a lesser extent Mexico (*F*_IS_ = 0.124—0.371) (Table 2).

**Table 2:** Genetic diversity estimates for sampled population of *Solanum rostratum* in China, the U.S.A. and Mexico.

**Region and Population**	***N***	***P***	***N*_*a*_ (range)**	***N*_*e*_ (± S.E.)**	**Allelic richness**	***H*_O_**	***H*_E_**	***F*_IS FSTAT_**	**Private Alleles**
**China**									
**BC**	24	0.60	1.90 (1–3)	1.39 (±0.16)	1.89	0.10	0.21	0.521***	1
**CY**	23	1.00	2.60 (2–4)	1.84 (±0.15)	2.60	0.36	0.42	0.174**	0
**WSL**	24	1.00	2.70 (2–5)	1.72 (±0.17)	2.69	0.28	0.37	0.280***	1
**MY**	24	1.00	2.80 (2–5)	1.65 (±0.14)	2.75	0.35	0.35	0.023	1
**TZ**	24	0.90	2.30 (1–3)	1.60 (±0.15)	2.28	0.28	0.32	0.164*	0
**Mean ± SE**	23.8 ± 0.20	0.90 ± 0.08	2.9 ± 0.16	1.6 ± 0.17	2.44± 0.16	0.27 ± 0.04	0.39 ± 0.04		0.6 ± 0.25
**U.S.A.**									
**HAY**	24	1.00	2.90 (2–5)	1.90 (±0.11)	2.89	0.27	0.46	0.433***	0
**BOT**	24	1.00	3.10 (2–5)	1.92 (±0.12)	3.09	0.42	0.45	0.104*	1
**ROL**	24	1.00	2.80 (2–6)	1.79 (±0.27)	2.79	0.22	0.36	0.415***	0
**CHE**	24	1.00	3.20 (2–7)	1.88 (±0.27)	3.18	0.29	0.4	0.303***	3
**WIC**	24	0.90	2.70 (1–6)	1.89 (±0.29)	2.69	0.29	0.39	0.269***	1
**Mean ± SE**	24 ± 0	0.98 ± 0.02	3.4 ± 0.09	1.9±0.10	2.93 ± 0.09	0.30 ± 0.03	0.45 ± 0.02		1.0 ± 0.55
**Mexico**									
**VDU**	19	1.00	4.10 (2–6)	2.39 (±0.26)	3.49	0.36	0.55	0.371***	7
**SLP**	15	0.90	4.00 (1–8)	2.24 (±0.22)	3.5	0.44	0.51	0.165**	4
**SLG**	22	1.00	4.70 (3–6)	2.54 (±0.33)	3.89	0.40	0.56	0.303***	5
**QSJ**	16	0.90	3.80 (1–8)	2.14 (±0.36)	3.12	0.40	0.44	0.124*	4
**TEM**	18	1.00	3.90 (2–8)	2.27 (±0.44)	3.42	0.40	0.45	0.137**	4
**Mean ± SE**	18 ± 1.22	0.98 ± 0.02	4.60 ± 0.16	2.30 ± 0.16	3.48 ± 0.12	0.39 ± 0.01	0.53 ± 0.02		4.80 ± 0.58
**Grand Mean ± S.E.**	21.93±0.84	0.95±0.03	3.17±0.20	1.94±0.08	2.95±0.13	0.32 ±0.02	0.42±0.02		2.20±0.56

*N* = number of individuals successfully genotyped; *P* = Proportion of polymorphic loci; *N*_a_ = Average number of alleles per locus; N_e_= Average number of effective alleles per locus; *R*_s_ = Allelic richness averaged across loci; *H*_O_ = Observed heterozygosity averaged across loci; *H*_E_ = Expected heterozygosity across loci (gene diversity); *F*_IS_ = inbreeding coefficient calculated in *F*-*STAT*, with 150000 randomizations, and a nominal level of significance of 1/1000. * P <0.05; ** *P*< 0.01; *** *P*<0.001; bold represents significance after correcting for multiple comparisons.

**Figure 2:**
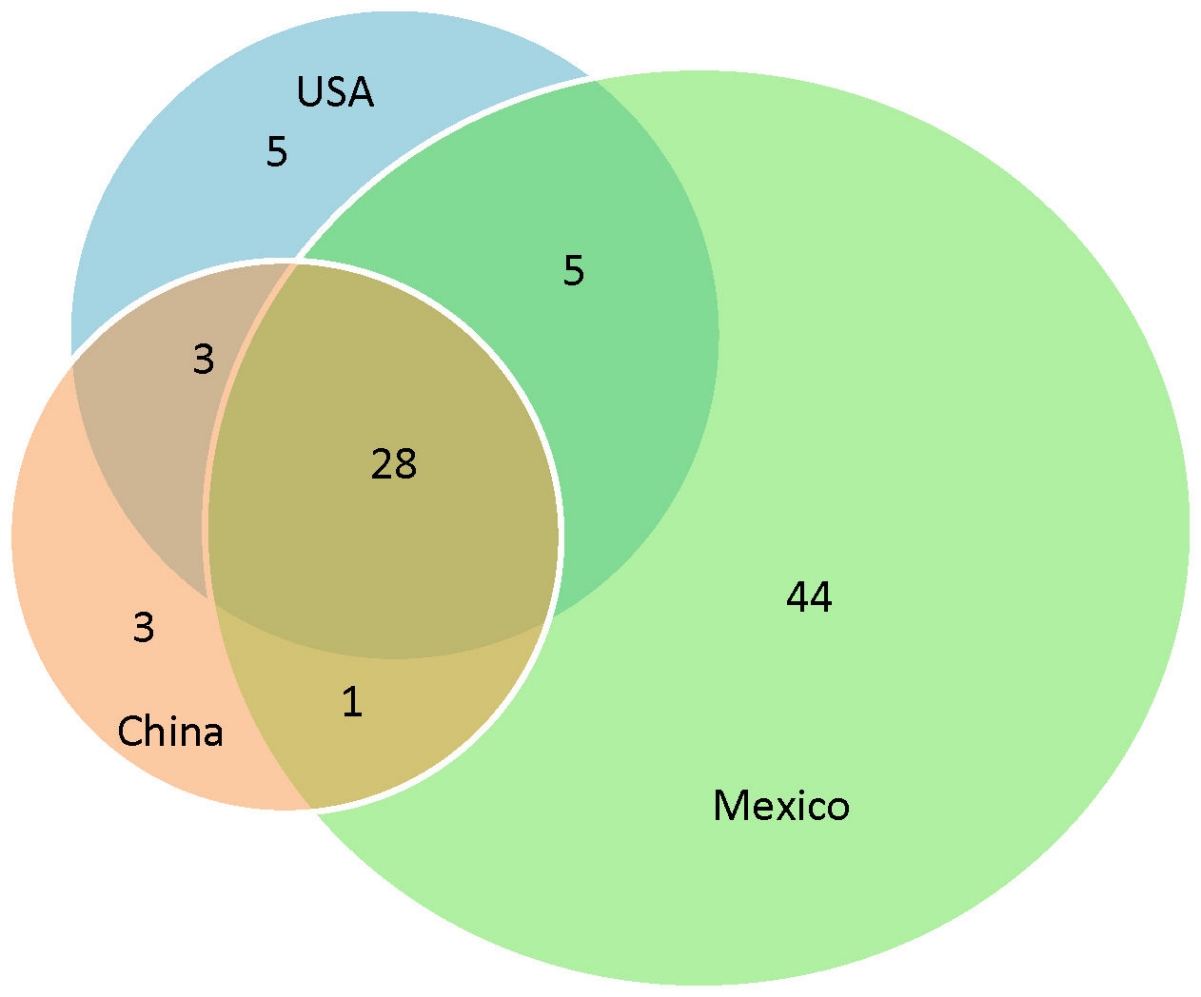
Venn diagram of the distribution of all distinct alleles across loci for the three geographic regions studied: Mexico, the U.S.A. and China. Overlapping areas (intersections) denote shared alleles between two or three regions.

A comparison across geographic regions indicated significant differences in the pattern of genetic variation. Allelic richness, observed heterozygosity (*H*_*O*_), and gene diversity (*H*_*E*_), were significantly higher in Mexico than in the U.S.A. and China (Table 3). Furthermore, the number of private alleles per region showed a strong contrast with nearly 50% (44/89) of alleles across loci being restricted to Mexico, and only between 3%—6% being found only in either the U.S.A. or China (Fig. 2). In contrast, neither the inbreeding coefficient (*F*_IS_) nor population differentiation (*F*_ST_) was statistically different between geographic regions (Table 3). The U.S.A. population had the lowest value of population differentiation (*F*_ST_ = 0.047), while Chinese populations had higher average genetic differentiation (*F*_ST_ = 0.267). Calculation of *F*_ST_ in China without the BC population reduced this value to *F*_ST_ =0.100, intermediate between the values observed in the U.S.A. and Mexico (*F*_ST_ = 0.159; Table 3). The mean number of private alleles of Mexican populations was nearly eight times larger than in China and four times larger than in the U.S.A. (Table 2). In summary, the highest levels of genetic diversity were observed in Mexican populations, while US populations had only marginally higher levels of diversity than China, while there were no statistically significant regional differences in either inbreeding within populations (*F*_IS_) or population differentiation (*F*_ST_) (Table 3).

**Table 3:** Comparison of genetic diversity across geographic regions.

	China	U.S.A.	Mexico	*P*-value
Allelic richness	2.162	2.56	3.483	0.001
*H*_O_	0.271	0.296	0.399	0.034
*H*_E_	0.343	0.425	0.521	0.001
*F*_IS_	0.209	0.302	0.234	0.548
*F*_ST_	0.267	0.047	0.159	0.167
	U.S.A.-China		Mexico	*P*-value
Allelic Richness:	2.361		3.483	0.001
*H*_O_	0.284		0.399	0.008
*H*_E_	0.384		0.521	0.002
*F*_IS_	0.261		0.234	0.623
*F*_ST_	0.171		0.159	0.544

P-value generated with 1000 randomizations of the data.

### Population Genetic Structure

Pairwise *F*_ST_ values, representing the degree of genetic differentiation between populations, ranged from 0.018 (CHE-HAY) to 0.399 (BC-TEM) in individual population pairs (Table S2). Among regions, the lowest average genetic differentiation between populations was observed in the U.S.A. (*F*_ST_=0.039, range 0.018–0.069), while Chinese populations had the highest average value (*F*_ST_=0.139, range 0.039–0.282). Mexican populations had an intermediate average *F*_ST_ (0.100, range 0.040–0.141). The high genetic differentiation observed among Chinese populations is due in part to the distinctiveness of one population, BC. This population has an average pairwise *F*_ST_ = 0.260 (0.224–0.282) with other Chinese populations and *F*_ST_=0.274 (0.224–0.399) when compared to all other 14 populations. Calculating pairwise *F*_ST_ without the BC population gives a Chinese average of *F*_ST_ = 0.059 ± 0.010, similar to the value observed for U.S.A. populations (Table S2). In pairwise comparisons between regions, average genetic differentiation with Mexican populations was *F*_ST_ = 0.241 ± 0.010 for China, and 0.187 ± 0.005 for the U.S.A. In contrast, average differentiation between U.S.A. and Chinese populations was lower (*F*_ST_ = 0.102 ± 0.005; *F*_ST_ = 0.062 ± 0.012 when excluding BC).

**Figure 3:**
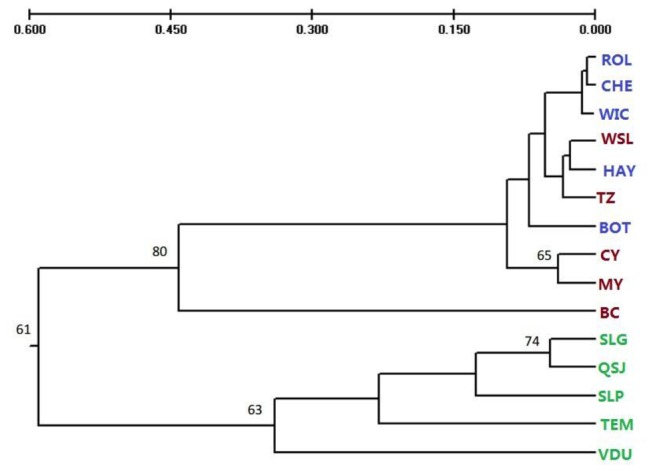
Unweighted pair group method with arithmetic mean (UPGMA) tree depicting the relationships between 15 *Solanum rostratum* populations from Mexico, the U.S.A. and China. The tree is based on the standardized genetic distances among populations (Nei's *D*_*S*_) calculated from a matrix of genotypes at 10 microsatellite loci. The numbers above the nodes represent percentage of bootstrap support (1000 replications). Only bootstrap support above 50% is shown. Population codes as in Table 1.

The UPGMA analysis calculated from Nei's pairwise genetic distance (*Ds*; Table S3) showed a split between Mexican and U.S.A./Chinese populations, which is supported by a 61% bootstrap value (Fig. 3). In contrast, Chinese and U.S.A. populations were hardly differentiated from each other, showing shallow branches with low bootstrap support (Fig. 3). The exception to this limited level of genetic distance among populations was the Chinese population BC, which formed a well-supported clade separate from the rest (Fig. 3). With the exception of BC, Mexican populations showed higher levels of genetic distance (deeper branches in Fig. 3; Table S3) than U.S.A./Chinese populations. This pattern of genetic variation is consistent with a recent divergence between the U.S.A. and most Chinese populations studied.

The results of the nested AMOVA indicated that the majority (70%) of genetic variation in *S. rostratum* is contained within populations (Table 4; *F*_ST_= 0.299). The proportion of genetic variance partitioned among populations within regions was 14% (*F*_SC_= 0.164), while differences among geographic regions explained 16% of the variation (*F*_CT_= 0.162). All variance components were statistically significant (*P*-values < 0.001; Table 4).

**Table 4:** Nested analysis of molecular variance (AMOVA) of 329 individuals in 15 populations of *Solanum rostratum* in three geographic regions: Mexico, the USA, and China.

**Source of variation**	***d.f.***	**Sum of squares**	**Variance components**	**% variation**	***P*-value**
**Among regions**	2	243.72	0.471	16.25	<0.001
**Among populations within regions**	12	231.72	0.394	13.59	<0.001
**Within populations**	643	1309.19	2.036	70.17	<0.001
**Total**	657	1784.64	2.901		

P-values were calculated using 1023 permutations, under the null hypothesis that a given variance component is equal to zero.

**Figure 4:**
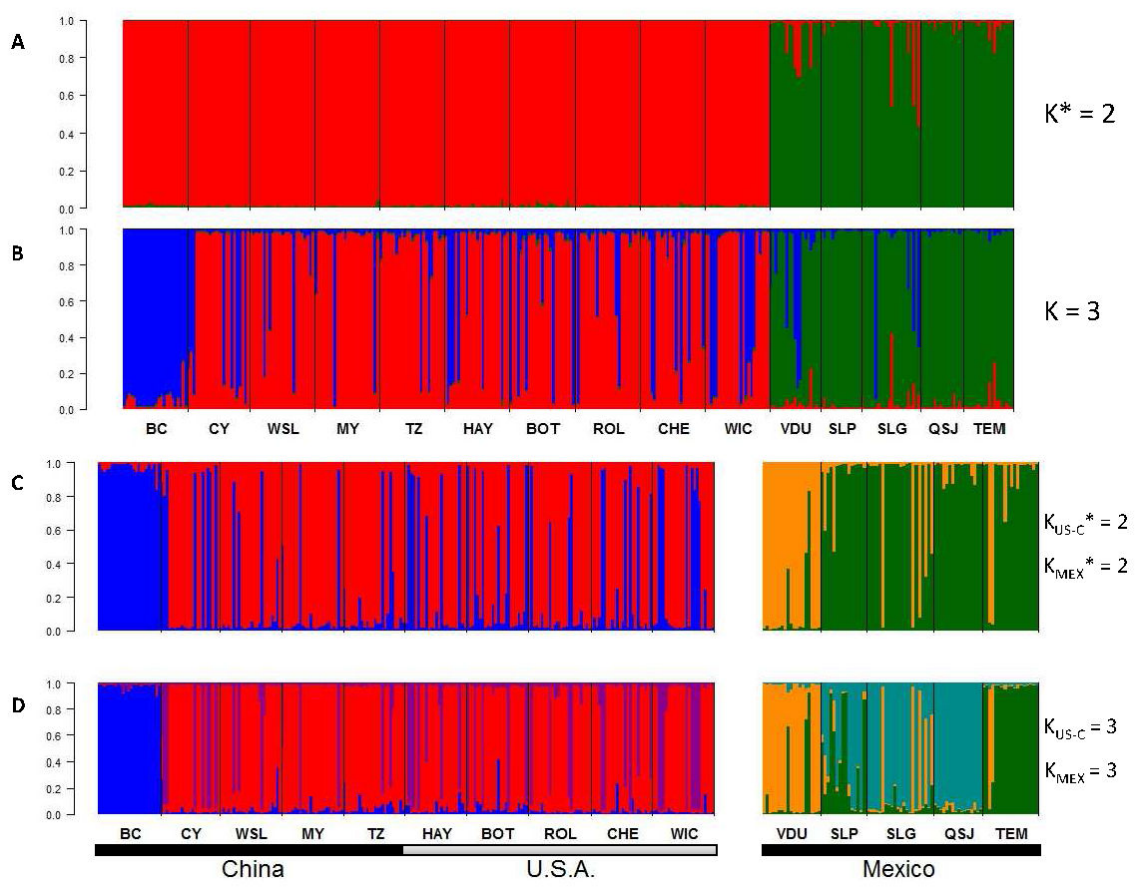
Posterior probability of cluster assignment in nested InStruct analyses of 329 *Solanum rostratum* individuals genotyped at 10 microsatellite loci across 15 populations in three geographic regions: Mexico, the U.S.A. and China. Each bar represents a single individual with populations separated by black lines and arranged from North to South within each region. Panel **A** depicts the uppermost level of population structure across regions. The optimal number of clusters (K* = 2) was calculated using Evanno’s et al. [31] *∆K* statistic. For illustration, panel **B** shows the assignment probabilities for *K* = 3. Panel **C** represent two separate analyses of either U.S.A./Chinese or Mexican populations; the optimal number of clusters [31] in each of these separate analyses was *K*_US-C_ * =*K*_US-C_ * = 2. Panel **D**, shows assignment probabilities for *K* = 3, for each separate analysis.

The InStruct analysis on the full data set found the strongest support for two clearly differentiated clusters (K* = 2) that corresponded to either Mexican or U.S.A./Chinese populations (Fig. 4). Increasing the number of clusters to *K* = 3 did not differentiate between US and Chinese populations, and instead individuals from populations in both geographic regions showed similar probabilities to be assigned to either of two clusters (Fig. 4). The BC population was the only one which was clearly assigned to a single of these clusters (Fig. 4). In the analysis within regions, the optimal number of clusters identified for Mexican populations was K* = 3 (Fig. 4). Populations VDU, QSJ and TEM showed little evidence of admixture at this level, while populations SLP and SLG contained individuals assigned to multiple clusters. The separate analysis of populations in the U.S.A. and China found an optimal number of clusters K* = 3 as well. Again, individuals in population BC were clearly differentiated from the rest forming a homogeneous and distinct cluster, but there was little evidence of population structure in other Chinese and U.S.A. populations (Figure 4).

**Figure 5:**
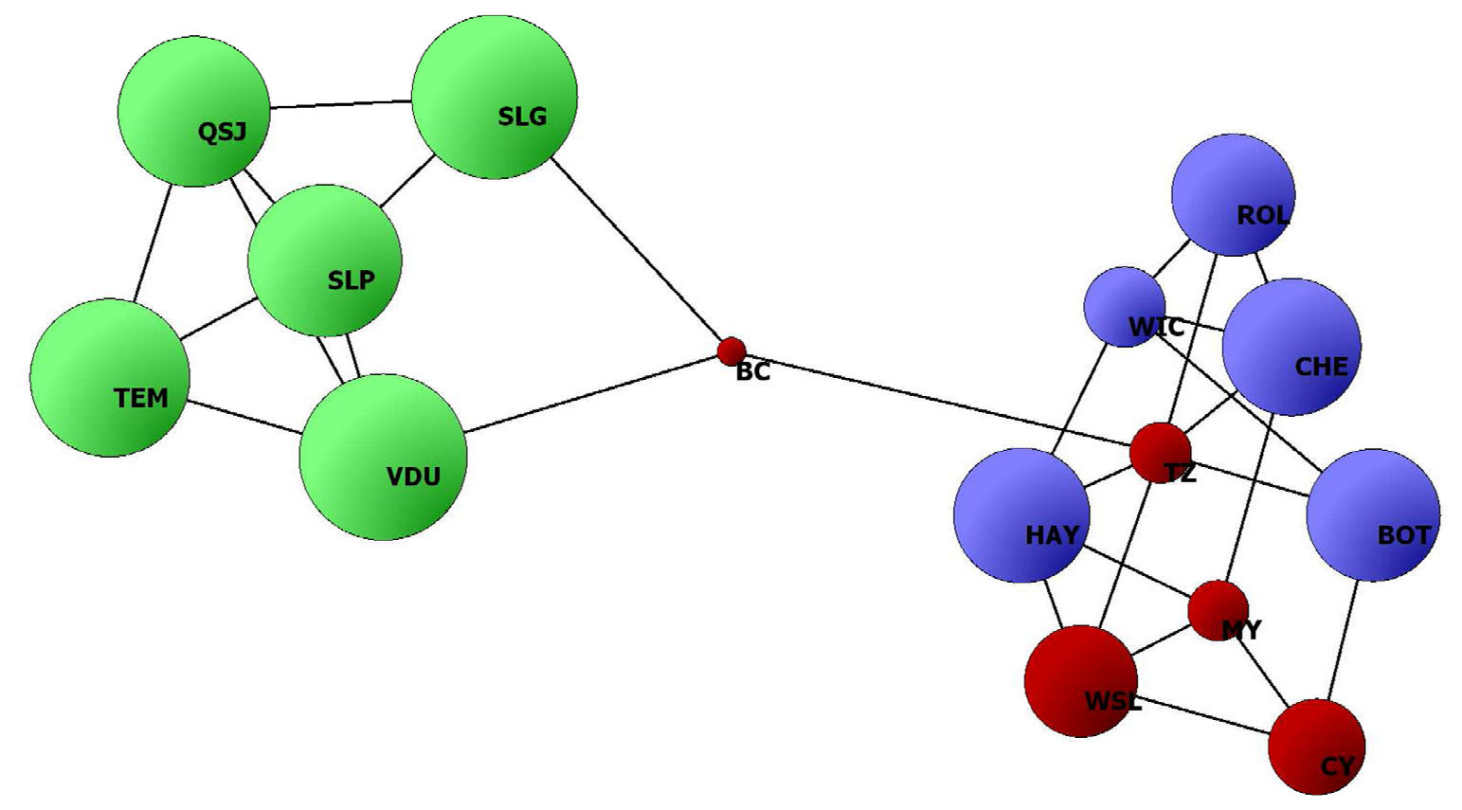
Network diagram of 15 populations of *Solanum rostratum* in Mexico (green circles), the U.S.A. (blue circles), and China (red circles) calculated in POPULATION GRAPH [32]. The diameter of each sphere is proportional to the amount of heterozygosity in each population. Black lines represent connections (edges) linking two populations.

In agreement with the UPGMA and InStruct results, the POPULATION GRAPH analysis showed two clearly differentiated groups composed of Mexican populations and China/U.S.A. populations (Fig. 5). The two groups were connected by two edges linking populations BC (China) and SLG and VDU (Mexico). The number of connections within each of these two groups was eight for the five Mexican populations, and 18 for the ten U.S.A./Chinese populations.

## Discussion

### Genetic diversity in native and introduced populations

We found the highest genetic diversity (allelic richness, gene diversity, and number of private alleles) among Mexican populations of *S. rostratum*, and significantly reduced diversity in U.S.A. and Chinese populations (Fig. 2). Whalen (1979) suggested that the highlands around Mexico City are probably the area of origin of *S. rostratum*, and indicated that this species is most phenotypically variable in central Mexico. The genetic data presented here supports the idea that Mexico is the core of diversity of *S. rostratum* and that populations in the U.S.A. and China have partly lost that diversity (Figure 2). The reduction in genetic diversity outside Mexico is consistent with a population bottleneck acting on introduced populations as it has been shown for invasive populations of other species (e.g.[5,35,36]). Our results indicate that other processes that can prevent and even reverse the loss of diversity in invasive populations such as multiple introductions [37] have not been strong enough to erase the signature of range expansion in *S. rostratum*.

 Although among populations we observed a wide range of variability in inbreeding rates (Table 2), we detected no dramatic differences in inbreeding across native and introduced regions (Table 3). This finding is perhaps surprising because successful long-distance dispersal may favour conditions leading to higher inbreeding including initially small population sizes, relatedness in the founding population, and selection for increased self-pollination [38-40]. In some cases, the most extreme form of inbreeding (self-pollination) is prevented in invasive species through self-incompatibility (e.g.[41,42]). However, as a self-compatible species, *S. rostratum* might rely on other aspects of its reproductive biology to maintain similar inbreeding levels in native and introduced populations. For example, the reproductive morphology of *S. rostratum*, which combines within-flower herkogamy, non-spontaneous pollen release requiring buzz-pollination, and enantiostyly may facilitate maintaining a relatively high outcrossing rate in introduced populations [22].

### Origin and genetic structure of the recent invasion to China

All of our analyses showed a stronger genetic affinity of Chinese populations with the U.S.A. than with Mexico, which demonstrates that invasive Chinese populations share a common origin with U.S.A. material. This common origin could be explained in at least two ways. First, Chinese populations could be directly derived from genetic stock in the U.S.A. region. Second, both U.S.A. and Chinese populations could have been independently derived from populations located elsewhere (e.g. unsampled populations in northern Mexico). Distinguishing between these two possibilities would require more extensive sampling throughout North America. However, given the residence time of *S. rostratum* in the U.S.A. (>130 years), there has been ample opportunities for the U.S.A. to act as the source material for the Chinese populations. 

The genetic differentiation between the Chinese populations at Baicheng in Jilin province (BC) raises the possibility that the invasion to China has occurred through multiple introductions. The BC population had the lowest genetic diversity and highest inbreeding coefficient of all populations, so part of this measured differentiation may be due to an extreme founding event [43]. However, another possibility is that the BC population comes from a separate introduction. There are some records of *S. rostratum* in the former Soviet Union [10], and is possible that BC was colonized from the north via Russia or eastern Mongolia. 

### Spread of introduced populations of *S. rostratum*

The relative rapid colonization of *S. rostratum* in northeast China raises the question as to how is it being dispersed. Using Chaoyang city, the first record of *S. rostratum* in China in 1981, as the focus for the spread of invasive populations and the location of the extant populations sampled here (at an approximate distance of 500km in both NE and SW directions), we can calculate that this species has dispersed at an average rate of 16km/year and probably much faster. This rate of dispersal is probably much too fast to be accounted naturally, especially given that this species lacks means for long-distance dispersal [10]. Earlier assertions (e.g.[44]) that the spiny fruits of this species could detach from the plant and become entangled with the fur of cattle and bison—incidentally one of the common names of *S. rostratum* is buffalo-bur—are incorrect. In reality, the fruits remain attached to the plant even after maturation and thus cannot be dispersed in isolation [10]. Instead, after the fruit matures, the spiny calyx surrounding the fruit partly opens and releases the small seeds (2.5mg ± 0.3 mg) which may travel short distances carried by wind or water [10]. In addition, Barrell ([45]; cited in [10]) also suggests that the congested habit of *S. rostratum* plants in the Great Plains could help them travel longer distances as tumble weeds. 

We believe that the rapid dispersal of *S. rostratum* in China has been facilitated by both massive change in land use and accidental human transport. As a weed of disturbed habitats and roadsides, *S. rostratum* may have taken advantage of the alteration of the landscape by people and cattle movement, and expanded its range by spreading through newly opened habitats. It is also likely that dispersal of *S. rostratum* is further facilitated by accidental transport in contaminated grain or forage as it often grows on the margins of crop fields, as well as by transfer of plant and soil waste. In China, some populations may have been also dispersed by seasonally flooded streams and transported as stowaways along train tracks and roads allowing them to quickly travel long distances. 

## Conclusions

The genetic data presented here demonstrates that the centre of diversity of *S. rostratum* is in Mexico, and shows that the colonization of this species to other areas has been accompanied by a loss of genetic diversity without an increase in the level of inbreeding. The invasion of *S. rostratum* to China has probably originated in sampled populations of the U.S.A. or closely related to them. The distinctiveness of the BC population from other Chinese populations indicates multiple introductions of *S. rostratum* into China.

## Supporting Information

Table S1 **Data per locus per population.** Obtained with Genalex.
(DOCX)

Table S2 **Pairwise *F*_ST_ values between 15 sampled populations of *Solanum rostratum*.**
(DOCX)

Table S3 **Nei’s Unbiased Identity (above diagonal) and Distance (*Ds*; below diagonal) among 15 sampled populations of *Solanum rostratum* derived from GenALEx.**
(DOCX)
