# Supplementary material for: Population Structure and Genetic Diversity of Native and Invasive Populations of Solanum rostratum (Solanaceae)
Source: PLoS One. 2013 Nov 5;8(11):e79807. doi: 10.1371/journal.pone.0079807 (PMC3818217; doi:10.1371/journal.pone.0079807)
Supplement: Table S1 — Data per locus per population. Obtained with Genalex. (DOCX) [file pone.0079807.s001.docx]

**Table S1.** Data per locus per population. Obtained with Genalex.

| Pop | Locus | N | N_a_ | N_e_ | H_o_ | H_e_ | UH_e_ | F_is_ |
| --- | --- | --- | --- | --- | --- | --- | --- | --- |
| BC | **Sr09** | 24 | 2 | 1.180 | 0 | 0.153 | 0.156 | 1.000 |
|  | **Sr18** | 24 | 1 | 1.000 | 0 | 0 | 0 | -- |
|  | **Sr30** | 24 | 1 | 1.000 | 0 | 0 | 0 | -- |
|  | **Sr06** | 24 | 3 | 1.892 | 0.500 | 0.471 | 0.481 | -0.061 |
|  | **Sr22** | 24 | 1 | 1.000 | 0 | 0 | 0 | -- |
|  | **Sr26** | 24 | 3 | 1.291 | 0 | 0.226 | 0.230 | 1.000 |
|  | **Sr05** | 24 | 1 | 1.000 | 0 | 0 | 0 | -- |
|  | **Sr21** | 24 | 2 | 2.000 | 0 | 0.500 | 0.511 | 1.000 |
|  | **Sr38** | 24 | 2 | 1.229 | 0.125 | 0.187 | 0.191 | 0.330 |
|  | **Sr12** | 24 | 3 | 2.323 | 0.417 | 0.569 | 0.582 | 0.268 |
| CY | **Sr09** | 23 | 2 | 1.091 | 0 | 0.083 | 0.085 | 1.000 |
|  | **Sr18** | 22 | 3 | 2.082 | 0.636 | 0.520 | 0.532 | -0.225 |
|  | **Sr30** | 23 | 3 | 1.365 | 0.217 | 0.267 | 0.273 | 0.187 |
|  | **Sr06** | 23 | 2 | 1.967 | 0.783 | 0.491 | 0.502 | -0.592 |
|  | **Sr22** | 23 | 2 | 1.873 | 0.652 | 0.466 | 0.476 | -0.400 |
|  | **Sr26** | 23 | 3 | 2.713 | 0.174 | 0.631 | 0.645 | 0.725 |
|  | **Sr05** | 22 | 3 | 1.449 | 0.364 | 0.310 | 0.317 | -0.173 |
|  | **Sr21** | 23 | 2 | 1.734 | 0 | 0.423 | 0.433 | 1.000 |
|  | **Sr38** | 23 | 2 | 1.830 | 0.522 | 0.454 | 0.464 | -0.150 |
|  | **Sr12** | 23 | 4 | 2.266 | 0.217 | 0.559 | 0.571 | 0.611 |
| WSL | **Sr09** | 24 | 2 | 1.087 | 0 | 0.080 | 0.082 | 1.000 |
|  | **Sr18** | 24 | 2 | 1.704 | 0.333 | 0.413 | 0.422 | 0.193 |
|  | **Sr30** | 24 | 2 | 1.438 | 0.208 | 0.305 | 0.311 | 0.316 |
|  | **Sr06** | 24 | 3 | 2.072 | 0.792 | 0.517 | 0.528 | -0.530 |
|  | **Sr22** | 24 | 2 | 1.229 | 0.208 | 0.187 | 0.191 | -0.116 |
|  | **Sr26** | 24 | 2 | 1.385 | 0.250 | 0.278 | 0.284 | 0.100 |
|  | **Sr05** | 24 | 4 | 2.577 | 0.333 | 0.612 | 0.625 | 0.455 |
|  | **Sr21** | 24 | 3 | 1.405 | 0 | 0.288 | 0.294 | 1.000 |
|  | **Sr38** | 24 | 2 | 1.753 | 0.375 | 0.430 | 0.439 | 0.127 |
|  | **Sr12** | 24 | 5 | 2.566 | 0.250 | 0.610 | 0.623 | 0.590 |
| MY | **Sr09** | 24 | 2 | 1.280 | 0 | 0.219 | 0.223 | 1.000 |
|  | **Sr18** | 24 | 2 | 1.997 | 0.542 | 0.499 | 0.510 | -0.085 |
|  | **Sr30** | 24 | 4 | 1.602 | 0.292 | 0.376 | 0.384 | 0.224 |
|  | **Sr06** | 24 | 3 | 2.076 | 0.958 | 0.518 | 0.529 | -0.849 |
|  | **Sr22** | 24 | 2 | 1.087 | 0.083 | 0.080 | 0.082 | -0.043 |
|  | **Sr26** | 24 | 3 | 2.032 | 0.375 | 0.508 | 0.519 | 0.262 |
|  | **Sr05** | 24 | 3 | 1.135 | 0.125 | 0.119 | 0.121 | -0.051 |
|  | **Sr21** | 24 | 2 | 1.180 | 0 | 0.153 | 0.156 | 1.000 |
|  | **Sr38** | 24 | 2 | 1.969 | 0.625 | 0.492 | 0.503 | -0.270 |
|  | **Sr12** | 24 | 5 | 2.182 | 0.500 | 0.542 | 0.553 | 0.077 |
| TZ | **Sr09** | 23 | 1 | 1.000 | 0 | 0 | 0 | -- |
|  | **Sr18** | 24 | 3 | 1.892 | 0.292 | 0.471 | 0.481 | 0.381 |
|  | **Sr30** | 24 | 2 | 1.986 | 0.333 | 0.497 | 0.507 | 0.329 |
|  | **Sr06** | 24 | 3 | 2.083 | 0.833 | 0.520 | 0.531 | -0.603 |
|  | **Sr22** | 24 | 2 | 1.133 | 0.125 | 0.117 | 0.120 | -0.067 |
|  | **Sr26** | 24 | 3 | 1.892 | 0.500 | 0.471 | 0.481 | -0.061 |
|  | **Sr05** | 24 | 3 | 2.268 | 0.500 | 0.559 | 0.571 | 0.106 |
|  | **Sr21** | 24 | 2 | 1.180 | 0 | 0.153 | 0.156 | 1.000 |
|  | **Sr38** | 24 | 2 | 1.438 | 0.125 | 0.305 | 0.311 | 0.590 |
|  | **Sr12** | 24 | 2 | 1.133 | 0.042 | 0.117 | 0.120 | 0.644 |
| HAY | **Sr09** | 24 | 2 | 1.704 | 0 | 0.413 | 0.422 | 1.000 |
|  | **Sr18** | 24 | 3 | 2.182 | 0.500 | 0.542 | 0.553 | 0.077 |
|  | **Sr30** | 24 | 2 | 1.969 | 0.375 | 0.492 | 0.503 | 0.238 |
|  | **Sr06** | 24 | 3 | 2.010 | 0.583 | 0.503 | 0.513 | -0.161 |
|  | **Sr22** | 24 | 3 | 1.410 | 0.167 | 0.291 | 0.297 | 0.427 |
|  | **Sr26** | 24 | 2 | 1.753 | 0.125 | 0.430 | 0.439 | 0.709 |
|  | **Sr05** | 24 | 5 | 2.521 | 0.542 | 0.603 | 0.616 | 0.102 |
|  | **Sr21** | 24 | 3 | 1.767 | 0 | 0.434 | 0.443 | 1.000 |
|  | **Sr38** | 24 | 2 | 1.438 | 0.208 | 0.305 | 0.311 | 0.316 |
|  | **Sr12** | 24 | 4 | 2.241 | 0.167 | 0.554 | 0.566 | 0.699 |
| BOT | **Sr09** | 23 | 2 | 1.091 | 0 | 0.083 | 0.085 | 1.000 |
|  | **Sr18** | 24 | 3 | 1.976 | 0.625 | 0.494 | 0.504 | -0.265 |
|  | **Sr30** | 24 | 2 | 1.969 | 0.375 | 0.492 | 0.503 | 0.238 |
|  | **Sr06** | 24 | 2 | 1.917 | 0.792 | 0.478 | 0.488 | -0.655 |
|  | **Sr22** | 24 | 4 | 2.472 | 0.667 | 0.595 | 0.608 | -0.120 |
|  | **Sr26** | 24 | 4 | 2.025 | 0.292 | 0.506 | 0.517 | 0.424 |
|  | **Sr05** | 24 | 5 | 2.190 | 0.500 | 0.543 | 0.555 | 0.080 |
|  | **Sr21** | 24 | 2 | 1.492 | 0 | 0.330 | 0.337 | 1.000 |
|  | **Sr38** | 24 | 3 | 2.153 | 0.458 | 0.536 | 0.547 | 0.144 |
|  | **Sr12** | 24 | 4 | 1.949 | 0.458 | 0.487 | 0.497 | 0.059 |
| ROL | **Sr09** | 24 | 2 | 1.087 | 0 | 0.080 | 0.082 | 1.000 |
|  | **Sr18** | 23 | 2 | 1.682 | 0.304 | 0.405 | 0.414 | 0.249 |
|  | **Sr30** | 24 | 2 | 1.917 | 0.208 | 0.478 | 0.488 | 0.564 |
|  | **Sr06** | 24 | 2 | 1.969 | 0.875 | 0.492 | 0.503 | -0.778 |
|  | **Sr22** | 24 | 3 | 1.684 | 0.250 | 0.406 | 0.415 | 0.385 |
|  | **Sr26** | 24 | 2 | 1.087 | 0 | 0.080 | 0.082 | 1.000 |
|  | **Sr05** | 24 | 4 | 1.829 | 0.208 | 0.453 | 0.463 | 0.540 |
|  | **Sr21** | 24 | 2 | 1.385 | 0 | 0.278 | 0.284 | 1.000 |
|  | **Sr38** | 24 | 3 | 1.237 | 0.042 | 0.192 | 0.196 | 0.783 |
|  | **Sr12** | 24 | 6 | 4.000 | 0.292 | 0.750 | 0.766 | 0.611 |
| CHE | **Sr09** | 24 | 2 | 1.600 | 0 | 0.375 | 0.383 | 1.000 |
|  | **Sr18** | 24 | 3 | 2.061 | 0.333 | 0.515 | 0.526 | 0.352 |
|  | **Sr30** | 24 | 2 | 1.969 | 0.375 | 0.492 | 0.503 | 0.238 |
|  | **Sr06** | 24 | 3 | 2.083 | 0.833 | 0.520 | 0.531 | -0.603 |
|  | **Sr22** | 24 | 3 | 1.237 | 0.208 | 0.192 | 0.196 | -0.086 |
|  | **Sr26** | 24 | 4 | 1.354 | 0.292 | 0.261 | 0.267 | -0.116 |
|  | **Sr05** | 24 | 4 | 1.682 | 0.250 | 0.405 | 0.414 | 0.383 |
|  | **Sr21** | 24 | 2 | 1.280 | 0 | 0.219 | 0.223 | 1.000 |
|  | **Sr38** | 24 | 2 | 1.385 | 0.167 | 0.278 | 0.284 | 0.400 |
|  | **Sr12** | 24 | 7 | 4.144 | 0.417 | 0.759 | 0.775 | 0.451 |
| WIC | **Sr09** | 24 | 2 | 1.704 | 0 | 0.413 | 0.422 | 1.000 |
|  | **Sr18** | 24 | 3 | 1.892 | 0.500 | 0.471 | 0.481 | -0.061 |
|  | **Sr30** | 24 | 2 | 1.946 | 0.417 | 0.486 | 0.496 | 0.143 |
|  | **Sr06** | 24 | 2 | 1.986 | 0.917 | 0.497 | 0.507 | -0.846 |
|  | **Sr22** | 24 | 4 | 1.772 | 0.417 | 0.436 | 0.445 | 0.044 |
|  | **Sr26** | 24 | 1 | 1.000 | 0 | 0 | 0 | -- |
|  | **Sr05** | 23 | 3 | 1.141 | 0.130 | 0.124 | 0.127 | -0.053 |
|  | **Sr21** | 24 | 2 | 1.800 | 0 | 0.444 | 0.454 | 1.000 |
|  | **Sr38** | 24 | 2 | 1.332 | 0.208 | 0.249 | 0.254 | 0.164 |
|  | **Sr12** | 24 | 6 | 4.347 | 0.333 | 0.770 | 0.786 | 0.567 |
| VDU | **Sr09** | 19 | 4 | 2.766 | 0.158 | 0.639 | 0.656 | 0.753 |
|  | **Sr18** | 19 | 6 | 2.124 | 0.526 | 0.529 | 0.543 | 0.005 |
|  | **Sr30** | 19 | 4 | 2.057 | 0.474 | 0.514 | 0.528 | 0.078 |
|  | **Sr06** | 19 | 4 | 2.292 | 0.368 | 0.564 | 0.579 | 0.346 |
|  | **Sr22** | 19 | 3 | 1.727 | 0.211 | 0.421 | 0.432 | 0.500 |
|  | **Sr26** | 19 | 5 | 2.344 | 0.526 | 0.573 | 0.589 | 0.082 |
|  | **Sr05** | 19 | 4 | 2.322 | 0.158 | 0.569 | 0.585 | 0.723 |
|  | **Sr21** | 16 | 2 | 1.519 | 0.313 | 0.342 | 0.353 | 0.086 |
|  | **Sr38** | 19 | 3 | 2.235 | 0.526 | 0.553 | 0.568 | 0.048 |
|  | **Sr12** | 19 | 6 | 4.513 | 0.316 | 0.778 | 0.799 | 0.594 |
| SLP | **Sr09** | 15 | 1 | 1.000 | 0 | 0 | 0 | -- |
|  | **Sr18** | 12 | 3 | 2.268 | 0.167 | 0.559 | 0.583 | 0.702 |
|  | **Sr30** | 15 | 5 | 1.875 | 0.600 | 0.467 | 0.483 | -0.286 |
|  | **Sr06** | 15 | 4 | 2.432 | 0.867 | 0.589 | 0.609 | -0.472 |
|  | **Sr22** | 14 | 6 | 2.292 | 0.500 | 0.564 | 0.585 | 0.113 |
|  | **Sr26** | 15 | 4 | 2.795 | 0.533 | 0.642 | 0.664 | 0.170 |
|  | **Sr05** | 13 | 8 | 3.521 | 0.769 | 0.716 | 0.745 | -0.074 |
|  | **Sr21** | 9 | 4 | 2.613 | 0.222 | 0.617 | 0.654 | 0.640 |
|  | **Sr38** | 13 | 2 | 1.742 | 0.462 | 0.426 | 0.443 | -0.083 |
|  | **Sr12** | 13 | 3 | 1.888 | 0.308 | 0.470 | 0.489 | 0.346 |
| SLG | **Sr09** | 22 | 4 | 1.689 | 0.045 | 0.408 | 0.418 | 0.889 |
|  | **Sr18** | 19 | 3 | 2.208 | 0.158 | 0.547 | 0.562 | 0.711 |
|  | **Sr30** | 22 | 5 | 1.462 | 0.364 | 0.316 | 0.323 | -0.150 |
|  | **Sr06** | 22 | 5 | 2.384 | 0.864 | 0.581 | 0.594 | -0.488 |
|  | **Sr22** | 22 | 5 | 2.225 | 0.136 | 0.551 | 0.563 | 0.752 |
|  | **Sr26** | 22 | 6 | 4.990 | 0.727 | 0.800 | 0.818 | 0.090 |
|  | **Sr05** | 19 | 5 | 3.099 | 0.421 | 0.677 | 0.696 | 0.378 |
|  | **Sr21** | 16 | 5 | 3.436 | 0.688 | 0.709 | 0.732 | 0.030 |
|  | **Sr38** | 22 | 4 | 1.816 | 0.318 | 0.449 | 0.460 | 0.292 |
|  | **Sr12** | 21 | 5 | 2.105 | 0.286 | 0.525 | 0.538 | 0.456 |
| QSJ | **Sr09** | 15 | 3 | 1.779 | 0.067 | 0.438 | 0.453 | 0.848 |
|  | **Sr18** | 16 | 2 | 2.000 | 0.750 | 0.500 | 0.516 | -0.500 |
|  | **Sr30** | 16 | 3 | 1.135 | 0.125 | 0.119 | 0.123 | -0.049 |
|  | **Sr06** | 16 | 2 | 1.992 | 0.813 | 0.498 | 0.514 | -0.631 |
|  | **Sr22** | 16 | 3 | 1.809 | 0.313 | 0.447 | 0.462 | 0.301 |
|  | **Sr26** | 16 | 5 | 2.876 | 0.688 | 0.652 | 0.673 | -0.054 |
|  | **Sr05** | 16 | 8 | 5.020 | 0.500 | 0.801 | 0.827 | 0.376 |
|  | **Sr21** | 16 | 7 | 1.875 | 0.250 | 0.467 | 0.482 | 0.464 |
|  | **Sr38** | 16 | 4 | 1.954 | 0.500 | 0.488 | 0.504 | -0.024 |
|  | **Sr12** | 16 | 1 | 1.000 | 0 | 0 | 0 | -- |
| TEM | **Sr09** | 18 | 3 | 1.780 | 0.111 | 0.438 | 0.451 | 0.746 |
|  | **Sr18** | 18 | 2 | 1.180 | 0.167 | 0.153 | 0.157 | -0.091 |
|  | **Sr30** | 18 | 3 | 1.674 | 0.389 | 0.403 | 0.414 | 0.034 |
|  | **Sr06** | 18 | 4 | 2.227 | 0.833 | 0.551 | 0.567 | -0.513 |
|  | **Sr22** | 18 | 5 | 1.608 | 0.389 | 0.378 | 0.389 | -0.029 |
|  | **Sr26** | 18 | 4 | 1.800 | 0.278 | 0.444 | 0.457 | 0.375 |
|  | **Sr05** | 18 | 8 | 5.635 | 0.833 | 0.823 | 0.846 | -0.013 |
|  | **Sr21** | 17 | 4 | 1.966 | 0.471 | 0.491 | 0.506 | 0.042 |
|  | **Sr38** | 18 | 2 | 1.117 | 0 | 0.105 | 0.108 | 1.000 |
|  | **Sr12** | 18 | 4 | 3.703 | 0.556 | 0.730 | 0.751 | 0.239 |
