# Supplementary material for: Population Structure and Genetic Diversity of Native and Invasive Populations of Solanum rostratum (Solanaceae)
Source: PLoS One. 2013 Nov 5;8(11):e79807. doi: 10.1371/journal.pone.0079807 (PMC3818217; doi:10.1371/journal.pone.0079807)
Supplement: Table S2 — Pairwise FST values between 15 sampled populations of Solanum rostratum. (DOCX) [file pone.0079807.s002.docx]

**Table S2.** Pairwise *F*_ST_ values between 15 sampled populations of *Solanum rostratum*.

|  | **China** | | | |  | **USA** | | | | |  | Mexico | | | | |
| --- | --- | --- | --- | --- | --- | --- | --- | --- | --- | --- | --- | --- | --- | --- | --- | --- |
| Population | CY | WSL | MY | TZ |  | HAY | BOT | ROL | CHE | WIC |  | VDU | SLP | SLG | QSJ | TEM |
| BC | 0.282 | 0.251 | 0.268 | 0.24 |  | 0.236 | 0.224 | 0.276 | 0.268 | 0.3 |  | 0.231 | 0.313 | 0.248 | 0.3 | 0.399 |
| CY |  | 0.041 | 0.043 | 0.09 |  | 0.051 | 0.044 | 0.076 | 0.069 | 0.079 |  | 0.193 | 0.253 | 0.208 | 0.256 | 0.229 |
| WSL |  |  | 0.055 | 0.039 |  | 0.033 | 0.048 | 0.044 | 0.038 | 0.067 |  | 0.194 | 0.253 | 0.204 | 0.243 | 0.224 |
| MY |  |  |  | 0.086 |  | 0.054 | 0.076 | 0.101 | 0.062 | 0.101 |  | 0.2 | 0.265 | 0.219 | 0.279 | 0.246 |
| TZ |  |  |  |  |  | 0.044 | 0.053 | 0.054 | 0.049 | 0.098 |  | 0.196 | 0.26 | 0.179 | 0.203 | 0.23 |
|  |  |  |  |  |  |  |  |  |  |  |  |  |  |  |  |  |
| HAY |  |  |  |  |  |  | 0.048 | 0.04 | 0.018 | 0.042 |  | 0.142 | 0.189 | 0.14 | 0.183 | 0.169 |
| BOT |  |  |  |  |  |  |  | 0.057 | 0.05 | 0.069 |  | 0.152 | 0.21 | 0.151 | 0.18 | 0.201 |
| ROL |  |  |  |  |  |  |  |  | 0.022 | 0.03 |  | 0.197 | 0.239 | 0.189 | 0.229 | 0.209 |
| CHE |  |  |  |  |  |  |  |  |  | 0.023 |  | 0.163 | 0.202 | 0.16 | 0.2 | 0.18 |
| WIC |  |  |  |  |  |  |  |  |  |  |  | 0.185 | 0.216 | 0.182 | 0.215 | 0.197 |
|  |  |  |  |  |  |  |  |  |  |  |  |  |  |  |  |  |
| VDU |  |  |  |  |  |  |  |  |  |  |  |  | 0.106 | 0.099 | 0.139 | 0.129 |
| SLP |  |  |  |  |  |  |  |  |  |  |  |  |  | 0.052 | 0.096 | 0.088 |
| SLG |  |  |  |  |  |  |  |  |  |  |  |  |  |  | 0.04 | 0.11 |
| QSJ |  |  |  |  |  |  |  |  |  |  |  |  |  |  |  | 0.141 |
