# Supplementary material for: Population Structure and Genetic Diversity of Native and Invasive Populations of Solanum rostratum (Solanaceae)
Source: PLoS One. 2013 Nov 5;8(11):e79807. doi: 10.1371/journal.pone.0079807 (PMC3818217; doi:10.1371/journal.pone.0079807)
Supplement: Table S3 — Nei’s Unbiased Identity (above diagonal) and Distance (Ds; below diagonal) among 15 sampled populations of Solanum rostratum derived from GenALEx. (DOCX) [file pone.0079807.s003.docx]

**Table S3.** Nei’s Unbiased Identity (above diagonal) and Distance (*Ds*; below diagonal) among 15 sampled populations of *Solanum rostratum* derived from GenALEx.

| Population | BC | CY | WSL | MY | TZ | HAY | BOT | ROL | CHE | WIC | VDU | SLP | SLG | QSJ | TEM |
| --- | --- | --- | --- | --- | --- | --- | --- | --- | --- | --- | --- | --- | --- | --- | --- |
| BC | — | 0.592 | 0.657 | 0.643 | 0.704 | 0.673 | 0.694 | 0.616 | 0.629 | 0.594 | 0.633 | 0.480 | 0.584 | 0.493 | 0.345 |
| CY | 0.524 | — | 0.951 | 0.962 | 0.892 | 0.939 | 0.938 | 0.901 | 0.915 | 0.898 | 0.526 | 0.422 | 0.508 | 0.474 | 0.517 |
| WSL | 0.419 | 0.050 | — | 0.932 | 0.963 | 0.974 | 0.939 | 0.954 | 0.961 | 0.928 | 0.568 | 0.469 | 0.559 | 0.534 | 0.560 |
| MY | 0.442 | 0.039 | 0.071 | — | 0.897 | 0.941 | 0.895 | 0.873 | 0.920 | 0.870 | 0.562 | 0.435 | 0.529 | 0.469 | 0.515 |
| TZ | 0.351 | 0.114 | 0.038 | 0.108 | — | 0.971 | 0.946 | 0.942 | 0.950 | 0.890 | 0.610 | 0.512 | 0.660 | 0.632 | 0.576 |
| HAY | 0.397 | 0.063 | 0.026 | 0.060 | 0.029 | — | 0.937 | 0.968 | 0.989 | 0.958 | 0.637 | 0.538 | 0.649 | 0.613 | 0.612 |
| BOT | 0.365 | 0.064 | 0.063 | 0.111 | 0.055 | 0.065 | — | 0.925 | 0.938 | 0.912 | 0.628 | 0.524 | 0.651 | 0.628 | 0.567 |
| ROL | 0.485 | 0.104 | 0.048 | 0.136 | 0.060 | 0.032 | 0.078 | — | 0.992 | 0.983 | 0.588 | 0.512 | 0.594 | 0.558 | 0.587 |
| CHE | 0.464 | 0.088 | 0.040 | 0.083 | 0.052 | 0.011 | 0.064 | 0.008 | — | 0.990 | 0.632 | 0.545 | 0.632 | 0.595 | 0.615 |
| WIC | 0.521 | 0.107 | 0.074 | 0.139 | 0.116 | 0.043 | 0.092 | 0.017 | 0.010 | — | 0.592 | 0.520 | 0.578 | 0.558 | 0.588 |
| VDU | 0.457 | 0.642 | 0.565 | 0.576 | 0.495 | 0.452 | 0.465 | 0.531 | 0.458 | 0.524 | — | 0.743 | 0.721 | 0.681 | 0.702 |
| SLP | 0.734 | 0.863 | 0.758 | 0.832 | 0.669 | 0.620 | 0.647 | 0.669 | 0.608 | 0.654 | 0.297 | — | 0.910 | 0.854 | 0.862 |
| SLG | 0.539 | 0.678 | 0.581 | 0.637 | 0.416 | 0.433 | 0.429 | 0.520 | 0.460 | 0.548 | 0.327 | 0.094 | — | 0.954 | 0.774 |
| QSJ | 0.708 | 0.746 | 0.627 | 0.758 | 0.458 | 0.490 | 0.465 | 0.583 | 0.519 | 0.584 | 0.384 | 0.158 | 0.047 | — | 0.755 |
| TEM | 1.064 | 0.659 | 0.579 | 0.663 | 0.552 | 0.491 | 0.567 | 0.533 | 0.486 | 0.531 | 0.353 | 0.148 | 0.257 | 0.282 | — |
